# Supplementary material for: HighDimMixedModels.jl: Robust high-dimensional mixed-effects models across omics data
Source: PLoS Comput Biol. 2025 Jan 13;21(1):e1012143. doi: 10.1371/journal.pcbi.1012143 (PMC11761659; doi:10.1371/journal.pcbi.1012143)
Supplement: S1 Appendix — (PDF) [file pcbi.1012143.s001.pdf]

S1 Appendix: Details on Coordinate Descent Algorithm

Due to their computational efficiency, coordinate descent algorithms are popular for solving penalized likelihood problems when the coordinate-wise optimization of the objective function has a closed-form solution. As discussed in [1], this happens to be the case for the objective function corresponding to penalized linear regression under the LASSO, but also under the SCAD, penalty. The coordinate descent approach was proposed for estimation of penalized linear mixed-effect model in [2] and [3], under the LASSO and SCAD penalties, respectively. This is the algorithm studied in this work and implemented in `HighDimMixedModels.jl` (Algorithm 1 in the main text). Here, we make some comments about whether this algorithm is actually maximizing the objective function Q_λ in Equation 2 of the main text, under the two penalties.

For the LASSO penalty, the well-known solution [4] to the univariate optimization problem in Line 8 of Algorithm 1 is

$$d^*_{LASSO}(g,h,\beta_j,\lambda) = \begin{cases} \frac{-g+\lambda}{h}, & \text{when } z < -\lambda \\ -\beta_j, & -\lambda \leq z \leq \lambda, \\ \frac{-g-\lambda}{h}, & \text{when } \lambda < z \end{cases},$$

where $z = h\beta_j - g = x_j^T V(\eta)^{-1}(y - X_{-j}\beta_{-j})$. Here, the notation $-j$ refers to the portion of the matrix/vector left over when the jth column/entry is removed. The update of β_j is thus

$$\beta_j^{\text{new}} = \beta_j + d^*_{LASSO}(g,h,\beta_j,\lambda) = \frac{S(z,\lambda)}{h} = \frac{\beta_j + d^*_{LASSO}(g,1,\beta_j,\lambda)}{h}, \tag{1}$$

where $S(\cdot,\cdot)$ denotes the soft-thresholding operation, $S(z,\lambda) = \text{sign}(z)(|z|-\lambda)_+$ In contrast, when we run Algorithm 1 with the SCAD penalty, at every update, we re-scale the input of the penalty function (line 7), so that we are no longer minimizing (with respect to the current coordinate while all others are fixed) our original objective function. Why do we do this?

When the input of the penalty function is rescaled with the SCAD penalty, the solution to the univariate optimization problem in Line 8 becomes

$$d^*_{\text{SCAD}}(g,h,\beta_j,\lambda) = \begin{cases} d^*_{LASSO}(g,h,\beta_j,\lambda), & \text{when } |z| < 2\lambda \\ \frac{S(z,a\lambda/(a-1))}{h(1-1/(a-1))} - \beta_j, & \text{when } 2\lambda \leq |z| \leq a\lambda \\ \frac{-g}{h}, & \text{when } a\lambda < |z| \end{cases},$$

This leads, in turn, to an update

$$\begin{aligned}\beta_j^{new} &= \beta_j + d^*_{SCAD}(g, h, \beta_j, \lambda)\\ &= \begin{cases} \frac{S(z,\lambda)}{h}, & \text{when } |z| < 2\lambda \\ \frac{S(z,a\lambda/(a-1))}{h(1-1/(a-1))}, & \text{when } 2\lambda \le |z| \le a\lambda \\ \frac{z}{h}, & \text{when } a\lambda < |z| \end{cases}\\ &= \frac{\beta_j + d^*_{SCAD}(g, 1, \beta_j, \lambda)}{h}\end{aligned}$$

In contrast, if we were to minimize the original objective function (with respect to the current coordinate while all others are fixed), we would obtain an update

$$\beta_j^{new} = \begin{cases} \frac{S(z,\lambda)}{h}, & \text{when } |z| < (h+1)\lambda \\ \frac{S(z,a\lambda/(a-1))}{h-1/(a-1)}, & \text{when } (h+1)\lambda \le |z| \le ah\lambda \\ \frac{z}{h}, & \text{when } ah\lambda < |z| \end{cases} \tag{2}$$

as long as $h > 1/(a-1)$. We found when we ran the algorithm with the SCAD penalty, we almost always have $|z| < (h+1)\lambda$, and thus, comparing Equations 1 and 2 we end up obtaining the same solutions under the SCAD penalty as under the LASSO if we do not adaptively rescale the input of the former penalty function. Further explanation for the rationale of this adaptive rescaling can be found in the discussion of coordinate descent for GLMs with non-convex penalties in [1]. We note that whereas we require rescaling in the mixed-effects context because the covariance matrix $V(\eta)$ changes over the course of the algorithm as we update the random effect parameters, it is needed for fitting generalized linear models (with only fixed effects) because of the changing weight matrix in the weighted least squares problem that results from quadratically approximating the log-likelihood at each iteration. In both cases, however, the consequence is the same, namely that $h = \frac{\partial^2(-\ell(\beta,\eta))}{\partial \beta_j^2}$ cannot be kept equal to a constant over the course of the algorithm, even if the covariates have been standardized prior to running the algorithm.

References

1. Breheny P, Huang J. Coordinate descent algorithms for nonconvex penalized regression, with applications to biological feature selection. The annals of applied statistics. 2011;5(1):232.

2. Schelldorfer J, Bühlmann P, van de Geer S. Estimation for high-dimensional linear mixed-effects models using ℓ1-penalization. Scandinavian Journal of Statistics. 2011;38(2):197–214.

3. Ghosh A, Thoresen M. Non-concave penalization in linear mixed-effect models and regularized selection of fixed effects. AStA Advances in Statistical Analysis. 2018;102:179–210.

4. Friedman J, Hastie T, Höfling H, Tibshirani R. Pathwise coordinate optimization. The annals of applied statistics. 2007;1(2):302–332.
