# Supplementary material for: HighDimMixedModels.jl: Robust high-dimensional mixed-effects models across omics data
Source: PLoS Comput Biol. 2025 Jan 13;21(1):e1012143. doi: 10.1371/journal.pcbi.1012143 (PMC11761659; doi:10.1371/journal.pcbi.1012143)
Supplement: S1 Tables — Table A. Random effect covariance matrices for the GWAS and gene expression simulations with q = 3 random effects. For simulations with 1 or 5 random effects, we specified a scalar covariance matrices, and the same scalar was chosen as the q = 3 case, i.e. 0.56. For fixed effect parameters, the non-zero components of the vector of fixed effect coefficients β was (1, 2, 4, 3, 3) in the settings with five non-zero effects and (1, 2, 4, 3, 3, −1, 5, −3, 2, 2) in the settings with ten. All other components were zero. All non-zero components appeared at the start of the vector in the order presented. The error variance σ2 was chosen to be 0.5 across all gene expression simulations. Table B. Random effect covariance matrices for the microbiome simulations with q = 3 random effects. For simulations with 1 or 5 random effects, we specified a scalar covariance matrices, and the same scalar was chosen as the q = 3 case, i.e. 0.56. The error variance and non-zero components of β were the same as in the gene expression and GWAS simulations (see caption in Table SA), with the exception that we added one additional non-zero component in β: a coefficient of −1 on the variable measured at the group level. Table C. Grid Search Parameters and Convergence Results for Simulation Settings. We used grid search to select λ for each simulated data set, and we searched over different grids depending on the simulation setting and the penalty being applied, as detailed in this table for the gene expression simulations. For the GWAS simulated data, we search over the interval 10 to 100 with increments of 1, and for the microbiome simulated data, we search over the same interval but with increments of 5 in all settings and penalties. These grids, as well as the convergence tolerance hyperparameter, were intentionally chosen so as to avoid convergence issues, since for each setting, a slightly different range of λ is necessary to avoid an interpolating solution. Despite these efforts, there were [file pcbi.1012143.s002.pdf]

S1 Tables

List of Tables

A Random effect covariance matrices for the gene expression and GWAS simulations with $q = 3$ random effects . . . 2
B Random effect covariance matrices for the microbiome simulations with $q = 3$ random effects 2
C Grid Search and Convergence Hyperparameters for all Simulation Settings . . . 3
D The results of true positive gene expression prediction under various conditions . . . 4
E The results of true positive GWAS prediction under various conditions. . . . 4
F The results of true positive microbiome prediction under various conditions . . . 4
G List of impactful genes in Riboflavin data . . . 5
H Taxonomic classification of OTUs selected by HighDimMM and their estimated regression coefficients . . . 6

Ψ_θ structure	Ψ_θ
scalar	$\begin{bmatrix} 0.56 & 0 & 0 \\ 0 & 0.56 & 0 \\ 0 & 0 & 0.56 \end{bmatrix}$
diagonal	$\begin{bmatrix} 3 & 0 & 0 \\ 0 & 3 & 0 \\ 0 & 0 & 2 \end{bmatrix}$
unstructured	$\begin{bmatrix} 3 & \sqrt{3} & -\sqrt{6} \\ \sqrt{3} & 4 & \sqrt{3}-\sqrt{2} \\ -\sqrt{6} & \sqrt{3}-\sqrt{2} & 5 \end{bmatrix}$

Table A: **Random effect covariance matrices for the gene expression and GWAS simulations with $q = 3$ random effects**. For simulations with 1 or 5 random effects, we specified a scalar covariance matrix, and the same scalar was chosen as the $q = 3$ case, i.e. 0.56. For fixed effect parameters, the non-zero components of the vector of fixed effect coefficients β was $(1, 2, 4, 3, 3)$ in the settings with five non-zero effects and $(1, 2, 4, 3, 3, -1, 5, -3, 2, 2)$ in the settings with ten. All other components were zero. All non-zero components appeared at the start of the vector in the order presented. The error variance σ^2 was chosen to be 0.5 across all gene expression simulations.

Ψ_θ structure	Ψ_θ
scalar	$\begin{bmatrix} 0.56 & 0 & 0 \\ 0 & 0.56 & 0 \\ 0 & 0 & 0.56 \end{bmatrix}$
diagonal	$\begin{bmatrix} 0.56 & 0 & 0 \\ 0 & 1 & 0 \\ 0 & 0 & 1 \end{bmatrix}$
unstructured	$\begin{bmatrix} 0.56 & 0.53 & 0.24 \\ 0.53 & 1.0 & -0.41 \\ 0.24 & -0.41 & 1.0 \end{bmatrix}$

Table B: **Random effect covariance matrices for the microbiome simulations with $q = 3$ random effects.** For simulations with 1 or 5 random effects, we specified a scalar covariance matrices, and the same scalar was chosen as the $q = 3$ case, i.e. 0.56. The error variance and non-zero components of β were the same as in the gene expression and GWAS simulations (see caption in Table A), with the exception that we added one additional non-zero component in β: a coefficient of -1 on the variable measured at the group level.

setting	penalty	$\lambda_{\min}$	$\lambda_{\max}$	increment	convergence tolerance
1	LASSO	40	60	5	1e-4
	SCAD	50	70	5	1e-4
2	LASSO	40	60	5	1e-4
	SCAD	50	70	5	1e-4
3	LASSO	50	70	5	1e-4
	SCAD	60	80	5	1e-4
4	LASSO	50	70	5	1e-4
	SCAD	60	80	5	1e-4
5	LASSO	45	55	1	1e-3
	SCAD	60	80	5	1e-4
6	LASSO	35	45	1	1e-3
	SCAD	60	80	5	1e-4
7	LASSO	35	45	1	1e-3
	SCAD	60	80	5	1e-4
8	LASSO	25	35	1	1e-3
	SCAD	60	80	5	1e-4
9	LASSO	30	40	1	1e-3
	SCAD	40	70	5	1e-4
10	LASSO	15	25	1	1e-3
	SCAD	35	55	1	1e-3
11	LASSO	15	35	1	5e-3
	SCAD	35	45	1	1e-3
12	LASSO	15	35	1	5e-3
	SCAD	25	45	1	5e-3
13	LASSO	30	45	3	1e-3
	SCAD	60	80	5	1e-4
14	LASSO	20	45	3	5e-3
	SCAD	50	80	5	1e-3

Table C: **Grid Search and Convergence Hyperparameters for all Simulation Settings.** We used grid search to select λ for each simulated data set, and we searched over different grids depending on the simulation setting and the penalty being applied, as detailed in this table for the gene expression simulations. For the GWAS simulated data, we search over the interval 10 to 100 with increments of 1, and for the microbiome simulated data, we search over the same interval but with increments of 5 in all settings and penalties. These grids, as well as the convergence tolerance hyperparameter, were intentionally chosen so as to avoid convergence issues, since for each setting, a slightly different range of λ is necessary to avoid an interpolating solution. Despite these efforts, there were certain gene expression simulation settings that proved especially difficult to fit with a LASSO penalty. In each of these difficult settings, there was at least one data set for which the coordinate gradient descent with a LASSO penalty converged to an interpolating solution for all of the λs that we searched over. These were setting 5 (one data set proved problematic), setting 6 (one data set proved problematic), setting 7 (three data sets proved problematic), and, most difficult of all, settings 8 and 10 (thirteen data sets proved problematic in each). Thus, while in general, the box plots in the main text show distributions over one hundred data sets each, for these particular settings with the LASSO penalty, the distributions depicted are over slightly fewer than one hundred data sets. When adopting the SCAD penalty, we were able to find at least one non-interpolating solution from the searched-over grid of λs for each data set (from each setting), so all boxplots depicting SCAD results are displaying a distribution over all one hundred data sets. Further, in the other two simulation domains (GWAS and microbiome), we were able to find at least one non-interpolating solution from the searched-over grid of λs in all settings under each attempted penalty.

penalty	predictor_cor	cov_str	$3 \leq$ TP<6 (%)	$6 \leq$ TP ≤ 9 (%)	TP = 10 (%)
LASSO	No	diagonal	0.61	0	0.39
LASSO	No	scalar	0.03	0	0.97
LASSO	No	unstructured	0	0	1
LASSO	Yes	diagonal	0.21	0.79	0
LASSO	Yes	scalar	0.63	0.26	0.1
LASSO	Yes	unstructured	0.06	0.73	0.21
SCAD	No	diagonal	0.02	0	0.98
SCAD	No	scalar	0	0	1
SCAD	No	unstructured	0.05	0.01	0.94
SCAD	Yes	diagonal	0.16	0	0.84
SCAD	Yes	scalar	0	0.01	0.99
SCAD	Yes	unstructured	0.06	0.03	0.91

Table D: **The results of true positive gene expression prediction under various conditions.** The columns indicate the type of penalty used (LASSO or SCAD), whether predictor variables are correlated ("Yes" or "No"), the covariance structure (diagonal, scalar, or unstructured), and the percentage of true positive among a total of 100 simulated data across different ranges of values.

penalty	cov_str	$3 \leq$ TP<6 (%)	$6 \leq$ TP ≤ 9 (%)	TP = 10 (%)
LASSO	diagonal	0.03	0.17	0.8
LASSO	scalar	0.01	0.11	0.88
LASSO	unstructured	0.11	0.11	0.78
SCAD	diagonal	0	0.18	0.82
SCAD	scalar	0	0.13	0.87
SCAD	unstructured	0.07	0.17	0.76

Table E: **The results of true positive GWAS prediction under various conditions.** The columns indicate the type of penalty used (LASSO or SCAD) and the covariance structure (diagonal, scalar, or unstructured), and the percentage of true positive among a total of 100 simulated data across different ranges of values.

otu_cor	cov_str	$3 \leq$ TP<6 (%)	$6 \leq$ TP ≤ 10 (%)	TP = 11 (%)
band	diagonal	0.23	0.02	0.75
band	scalar	0.03	0	0.97
band	unstructured	0.15	0.01	0.84
scale_free	diagonal	0.01	0	0.99
scale_free	scalar	0.01	0	0.99
scale_free	unstructured	0.01	0	0.99

Table F: **The results of true positive microbiome prediction under various conditions.** The columns indicate the structure of the network (band or scale_free) and the covariance structure (diagonal, scalar, or unstructured), along with the percentage of true positive predictions among a total of 100 simulated data across different ranges of values.

lmmlasso	lmmscad	HighDimMixedModels (SCAD)
GAP229-F	*LCTE*	*YFJD*
LCTE	***LYSC***	*YTOI*
PRIA	*RIBR*	***LYSC***
ssuA	***TUAH***	*METK*
ssuC	*YCLN*	***TUAH***
TUAH	***YDDK***	*UVRB*
YCDH	*YKPC*	*YCGP*
YDCP	*YOCR*	*YDBM*
YDDK	***YURQ***	*YFKE*
YEEI	***YXLD***	*YLMA*
YFJC		*YNEI*
YHFU		*YTXM*
YHZA		*YUBB*
YKBA		***YURQ***
YKPC		*YUSY*
YLOM		***YXLD***
YOCJ		***YDDK***
YOCR		
YQGH		
YQJG		
YTFP		
YTOA		
YTRP		
YURR		
YVRK		
YXKC		
YXLD		

Table G: **List of impactful genes identified when fitting model to Riboflvain data using our implementation (HighDimMixedModels) versus R implementations (lmmlasso and lmmscad)** The intersection of genes between the implementations is highlighted in bold.

OTU index	Family	Genus	Species	Regression coefficient estimate
365	Clostridiaceae	Clostridium	hiranonis	-0.9811
839	Lachnospiraceae	Blautia	unclassified	0.0547
9	Bifidobacteriaceae	Bifidobacterium	unclassified	-0.0428
1224	Streptococcaceae	Streptococcus	unclassified	-0.0347
125	Veillonellaceae	Veillonella	dispar	-0.0147
23	Peptostreptococcaceae	unclassified	unclassified	0.006
140	unclassified	unclassified	unclassified	-0.005
110	Micrococcaceae	Rothia	mucilaginosa	-0.0006
345	Ruminococcaceae	Faecalibacterium	prausnitzii	0.0001

Table H: **Taxonomic classification of OTUs selected by HighDimMM and their estimated regression coefficients.** *Clostridium hiranonis* was also assigned a quadratic term, but we show here only the coefficient on the linear term. The OTUs are ordered by the magnitude of their coefficient.
