# Supplementary material for: HighDimMixedModels.jl: Robust high-dimensional mixed-effects models across omics data
Source: PLoS Comput Biol. 2025 Jan 13;21(1):e1012143. doi: 10.1371/journal.pcbi.1012143 (PMC11761659; doi:10.1371/journal.pcbi.1012143)
Supplement: Attachment — Submitted filename: Reviewer_HighDimMixedModels.jl.pdf [file pcbi.1012143.s018.pdf]

23 October 2024

Re: HighDimMixedModels.jl: Robust High Dimensional Mixed Models across Omics Data

To editor,

We have addressed all of the reviewers' comments in this new version (changes highlighted in blue). In the preparation of this revision, we identified some method details (such as details on the coordinate descent algorithm) that were omitted in the initial version submitted, and that we believe will greatly enhance the manuscript. Thus, in addition to the reviewers' comments, we have expanded some details on the Methods section. Last, based on a reviewer's comment, we changed the order of the sections to present Methods first, and then Results which is not aligned with the journal's template, but we believe that it allows the manuscript to be clearer. Below, we provide a point-by-point response to each comment.

Responses to the Referees (a point-to-point reply to referees)

Reviewer Comments:

Reviewer 1

In this manuscript, the authors introduced a Julia package for high-dimensional mixed-effects model variable selection and did a simulation study to evaluate the program's performance on various omics data. More importantly, the manuscript offers a quite inclusive review of the existing methods and publicly available packages/codes for high-dimensional mixed-effects model variable selection, which can be very helpful for researchers who work with high-dimensional data. I can see the manuscript has great value in application and could be very appealing for readers in the computational biology field. Please see below for issues I think the authors should address before publication and my suggestions:

1- The most unconventional feature I found of this manuscript is that the method and simulation settings section are put after the results section. This arrangement is probably common for this journal, but for a simulation study paper I found it quite inconvenient as the readers are given the results before they have a chance to understand the simulation setting. Without the knowledge of how the data is simulated, it is challenging for readers to interpret the simulation results. I will leave it to the editor to decide the optimal arrangement, but I personally recommend presenting the simulation setting before the results.	Thank you for this valuable feedback about the organization of the manuscript. We agree that by placing the description of the simulation settings at the end of the manuscript, it's very difficult to make sense of and interpret the results of the simulations when reading the manuscript in a linear order.<br><br>We used this arrangement because it is the template provided by the journal.<br>However, based on your suggestion, we have changed the order of the sections to present the Methods first, followed by the Results.<br><br>Second, we have added a new subsection,

	Subsection 3.1: Characteristics of Omics Data Sets, to directly precede the simulation results. This section also addresses the point you raise in your next suggestion: it provides a description of the unique characteristic of each of the different omics data types we focus on in the paper.
2- Although the target audience of this paper is computational biologist, but I believe it can be generalized to researchers in other fields as high-dimensional clustered data can be seen in many fields. It would be very helpful to give a brief introduction on each omics data type being evaluated in the simulations in the introduction section or in the simulation setting section, such as "what are the characteristics of the gene expression data/GWAS, how are they different from other high-dimensional data and what are the challenges it posed to the other methods" etc. For readers outside the computational biology field the concept of omics data maybe very confusing and have troubles understanding the simulation results because they don't know what the data look like (especially the simulation setting section is present in the end of the paper). A brief introduction of these data could help these readers to better understand and make connection between the evaluated data and the data in their own research.	Thank you for this thoughtful feedback. We appreciate your suggestion to provide more context on the types of omics data used in our simulations, as this would indeed make the paper more accessible to readers outside the field of computational biology.<br><br>As mentioned above, we've added a section called "Characteristics of Omics Data Sets" to the manuscript. We hope this section clarifies what the unique characteristics of the different omics data types we focus on in the paper are.
3- Effect estimation results are present in a peculiar way. Usually estimation results are present in bias and standard deviation from all estimated coefficients, but in this paper only the estimations of one penalized coefficient and one unpenalized coefficient are shown in setting. The results of only two coefficients estimation are not very informative about the program's performance. Could the authors provide a more inclusive evaluation or elaborate on what is special about the chosen two coefficients that only their estimations are displayed?	Thank you for pointing out this unusual feature of our presentation. The idea was that the estimates of all penalized coefficients share a common or similar distribution and the estimates of all unpenalized coefficients share a common or similar distribution, so in order to show results for many different simulation settings in the same figure, we chose β_3 as a representative of the unpenalized coefficients and β_4 for the penalized.<br>However, your critique makes us realize that it may be nice for the reader to see a plot with all coefficients at once, so we now include such a plot for the gene expression simulations (Figure 2) in our manuscript.

**Minor issues:**	
1- Line 62 on page 2 “For example, if the number of covariates exceeds the total sample size, it becomes possible to find fixed effect regression coefficients that interpolate the data, ...”, should “becomes possible” be “becomes impossible”?	“Becomes possible” was indeed correct. What we mean by this is that with enough parameters, you can perfectly fit the data, which is the reason why maximum likelihood estimation in the high dimensional setting results in over-fitting. For clarity, we’ve revised the wording to the following: “Unfortunately, standard maximum and restricted maximum likelihood estimation of the parameters of the mixed-effects models deteriorates in the high dimensional setting. In the extreme case, if the number of covariates exceeds the total sample size, there are no degrees of freedom to form the restricted likelihood, and maximum likelihood estimation entails interpolating the data with the fixed effects and sending the error variance to 0” (lines 54-58 in the new manuscript).
2- Line 394 on page 14, the notation for iteration is k, but k has also been used as the index for fixed effects coefficients beta k. Please consider changing the notation to avoid confusion.	Thanks for pointing out this notational issue. In the resubmitted manuscript, we now use j alone to index beta (see, e.g., line 139) and retain k as the iteration index (as in Algorithm 1 on page 5).
3- Reference 20 and 21 are duplicates.	Thanks for pointing this out, we’ve eliminated the duplicated reference. Note that references 19 and 20 are now to works from the same author, but the first is citing their R package and the second their paper.

Reviewer #2: Yes: Tyrone Chen

**Major comments:**	
1- Benchmarking of the various omics studies is carried out, but addressed individually. It would be useful for the reader to see a combined summary table or figure indicating the performance of the HigDimMixedModels approach against the results/models of each study featured in the manuscript.	Thank you for your insightful comment. In response, we have added a new subsection titled "3.3 Application to Real Data" in the main paper. This subsection includes a new figure (Figure 6) that summarizes the standard deviations (SD) of non-zero estimates for penalized coefficients across all the omics data sets studied (gene expression, GWAS, and microbiome).
Line 39-40<br><br>“We provide a table summarising existing methods”<br><br>It is possible that I missed it, but it seems that this table is missing from the supplementary material at this time? A direct reference to the table number would also be useful.	Thank you for bringing this to our attention! We’ve added the missing link (in lines 34 and 99)
**Minor comments:**	
1- Scripts, data, documentation and results are available and well-organised, which streamlines the review process, boosts reproducibility and software use. However, it would be useful for the authors to provide an estimate of compute resource use and time for a given dataset (e.g. those outlined in the manuscript and documentation).	Thank you for this suggestion. We agree that computation time is essential information for those who want to use our software, and failure to include this information was an oversight on our part. We’ve added information on the time it takes for our algorithm to converge when fitting each of the real data sets in Table 3.
2- The scope of this package and study is focused on analysing single-omics data.	Thank you for bringing this up. We agree that recent studies involve more than one data type

However, many studies now incorporate multi-omics data analyses instead of single-omics analyses. If I understand correctly, it appears that this work may complement multi-omics studies by standardising data in a generic way though before multi-omics data integration (eg by using the mixOmics package). Can the authors discuss the potential use of this method for this application?	and while we believe that our implementation will suit a multi-omics dataset, an extensive test is beyond the scope of the current project and will be considered as future work.
4- The discussion section implies that the authors intend to refine and expand their work. For the future, it would be useful to add biological examples (e.g. those in the manuscript) to the documentation directly covering multiple use case scenarios. Currently, the documentation has an example of its use on a small non-biological dataset, although this is understandable for testing purposes.	This is a great idea. We have added a new high dimensional example to the documentation https://solislemuslab.github.io/HighDimMixedModels.jl/dev/man/example/. However, this is a simulated example and not one of the omics data sets analyzed in our paper. We plan to add an example with a real omics data set to the documentation in the future.
Line 362<br><br>Binary or count data is common in biology and is often transformed into a continuous distribution for analysis, for example in the case of RNA-Seq data. The authors may be interested in adding some comments on the potential feasibility of adapting this method for non-continuous data.	Thank you for this insightful comment. It is indeed very important to handle these types of responses, as binary and count data are common in omics studies. We discuss this in the last paragraph of the Discussion section in our manuscript and comment on the need to study the empirical performance of the algorithms proposed in references [30], [68], and [60] for fitting high dimensional generalized linear mixed models.
