# Supplementary material for: HighDimMixedModels.jl: Robust high-dimensional mixed-effects models across omics data
Source: PLoS Comput Biol. 2025 Jan 13;21(1):e1012143. doi: 10.1371/journal.pcbi.1012143 (PMC11761659; doi:10.1371/journal.pcbi.1012143)
Supplement: Attachment — Submitted filename: review report.docx [file pcbi.1012143.s017.docx]

Robust High Dimensional Mixed Models across Omics Data

In this manuscript, the authors introduced a Julia package for high-dimensional mixed-effects model variable selection and did a simulation study to evaluate the program’s performance on various omics data. More importantly, the manuscript offers a quite inclusive review of the existing methods and publicly available packages/codes for high-dimensional mixed-effects model variable selection, which can be very helpful for researchers who work with high-dimensional data. I can see the manuscript has great value in application and could be very appealing for readers in the computational biology field. Please see below for issues I think the authors should address before publication and my suggestions:

1. The most unconventional feature I found of this manuscript is that the method and simulation settings section are put after the results section. This arrangement is probably common for this journal, but for a simulation study paper I found it quite inconvenient as the readers are given the results before they have a chance to understand the simulation setting. Without the knowledge of how the data is simulated, it is challenging for readers to interpret the simulation results. I will leave it to the editor to decide the optimal arrangement, but I personally recommend presenting the simulation setting before the results.

2. Although the target audience of this paper is computational biologist, but I believe it can be generalized to researchers in other fields as high-dimensional clustered data can be seen in many fields. It would be very helpful to give a brief introduction on each omics data type being evaluated in the simulations in the introduction section or in the simulation setting section, such as “what are the characteristics of the gene expression data/GWAS, how are they different from other high-dimensional data and what are the challenges it posed to the other methods” etc. For readers outside the computational biology field the concept of omics data maybe very confusing and have troubles understanding the simulation results because they don’t know what the data look like (especially the simulation setting section is present in the end of the paper). A brief introduction of these data could help these readers to better understand and make connection between the evaluated data and the data in their own research.
3. Effect estimation results are present in a peculiar way. Usually estimation results are present in bias and standard deviation from all estimated coefficients, but in this paper only the estimations of one penalized coefficient and one unpenalized coefficient are shown in setting. The results of only two coefficients estimation are not very informative about the program’s performance. Could the authors provide a more inclusive evaluation or elaborate on what is special about the chosen two coefficients that only their estimations are displayed?

Minor issues:

1. Line 62 on page 2 “For example, if the number of covariates exceeds the total sample size, it becomes possible to find fixed effect regression coefficients that interpolate the data, ...”, should “becomes possible” be “becomes impossible”?
2. Line 394 on page 14, the notation for iteration is k, but k has also been used as the index for fixed effects coefficients beta k. Please consider changing the notation to avoid confusion.
3. Reference 20 and 21 are duplicates.
